# Allosteric inhibition of carnosinase (CN1) by inducing a conformational shift

**DOI:** 10.1080/14756366.2017.1355793

**Published:** 2017-08-04

**Authors:** Verena Peters, Claus P. Schmitt, Tim Weigand, Kristina Klingbeil, Christian Thiel, Antje van den Berg, Vittorio Calabrese, Peter Nawroth, Thomas Fleming, Elisabete Forsberg, Andreas H. Wagner, Markus Hecker, Giulio Vistoli

**Affiliations:** a Centre for Paediatric and Adolescent Medicine, University of Heidelberg, Heidelberg, Germany;; b Department of Biomedical and Biotechnological Sciences, School of Medicine, University of Catania, Catania, Italy;; c Department of Internal Medicine, University Heidelberg, Heidelberg, Germany;; d The Rolf Luft Center Research Center for Diabetes and Endocrinology, Karolinska Institutet, Stockholm, Sweden;; e Institute for Physiology and Pathophysiology, University Heidelberg, Heidelberg, Germany;; f Department of Pharmaceutical Sciences, Università degli Studi di Milano, Milan, Italy

**Keywords:** Carnosinase 1 activity, CN1, allosteric inhibition, glutathione, N-acetylcysteine, diabetes

## Abstract

In humans, low serum carnosinase (CN1) activity protects patients with type 2 diabetes from diabetic nephropathy. We now characterized the interaction of thiol-containing compounds with CN1 cysteine residue at position 102, which is important for CN1 activity. Reduced glutathione (GSH), N-acetylcysteine and cysteine (3.2 ± 0.4, 2.0 ± 0.3, 1.6 ± 0.2 µmol/mg/h/mM; *p* < .05) lowered dose-dependently recombinant CN1 (rCN1) efficiency (5.2 ± 0.2 µmol/mg/h/mM) and normalized increased CN1 activity renal tissue samples of diabetic mice. Inhibition was allosteric. Substitution of rCN1 cysteine residues at position 102 (Mut1^C102S^) and 229 (Mut2^C229S^) revealed that only cysteine-102 is influenced by cysteinylation. Molecular dynamic simulation confirmed a conformational rearrangement of negatively charged residues surrounding the zinc ions causing a partial shift of the carnosine ammonium head and resulting in a less effective pose of the substrate within the catalytic cavity and decreased activity. Cysteine-compounds influence the dynamic behaviour of CN1 and therefore present a promising option for the treatment of diabetes.

## Introduction

Carnosinase (CN1, EC 3.4.13.20) plays an important role in the development of nephropathy in diabetic patients. CN1 is encoded by the *CNDP1* gene^1^ and susceptibility to diabetic nephropathy (DN) in patients with diabetes mellitus type II is associated with a polymorphism in the *CNDP1* gene^2^. The shortest allelic form, the so-called *CNDP1* “Mannheim allele” (D18S880, homozygosity for the five-leucine allele), is associated with lower serum CN1 activities and was found to be associated with a lower prevalence of DN^2^^,^^3^. CN1 belongs to the M20 family of metalloproteases and cleaves histidine-containing dipeptides, such as carnosine (β-alanyl-l-histidine) and anserine (β-alanyl-l-1-methylhistidine)^1^. Carnosine scavenges carbonyls^4–6^, inhibits glycation^7^ and acts as ACE inhibitor^8^^,^^9^. Its function as antioxidant is debated^10–14^. It restores erythrocyte deformability^15^, inhibits cellular senescence^16^^,^^17^ as well as the production of matrix proteins such as fibronectin and collagen type VI by podocytes^2^. Carnosine is actively absorbed in the gastro-intestinal tract via the hPepT1 transporter and rapidly hydrolysed by CN1 in plasma, which precludes therapeutic administration of carnosine in humans, e.g. for treatment of diabetic sequelae^18^^,^^19^. Development of carnosine derivatives resistant to CN1 hydrolysis has become the subject of emerging interest in recent years^20^. In rodents, no serum CN1 is present and carnosine supplementation of diabetic mice increases serum and tissue carnosine levels and mitigates DN, reduces renal vasculopathy^21^, normalizes vascular permeability^21^, improves wound healing^22^ and decreases insulin growth factor binding protein-1 (IGFBP1) production through suppression of HIF-1α, and improves glucose homeostasis^23^. Moreover, in streptozotocin-induced diabetic rats, carnosine prevents apoptosis of glomerular cells, podocyte loss^24^^,^^25^, and vascular damage^26^.

In contrast to rodents, dietary supply of carnosine does not increase systemic histidine dipeptide concentrations due to rapid degradation by CN1 (19). An alternative approach to increase tissue carnosine concentrations and associated protective actions is pharmacological inhibition of CN1 activity. CN1 is a homodimer *in vitro*^1^, but present as a monomer and a dimer *in vivo*^27^. Each monomer consists of a catalytic domain and a dimerization domain with the catalytic domain featuring a dinuclear Zn^2+^-containing active site^28^. The age-dependent increase of serum CN1 activity in children and adults^29^ is not caused by higher CN1 concentrations but due to allosteric conformational changes^27^^,^^30^. Carnosine degradation rate by CN1 is also affected by substrate inhibition of the CN1 substrates anserine^31^ and homocarnosine^27^. Molecular dynamic (MD) simulations demonstrated that the higher affinity of homocarnosine is based, at least in part, on more extensive interactions inside the active site of CN1^28^. Cysteine substitutions in the recombinant CN1 indicated the relevance of the cysteine residue at position 102 (Cys102) for its catalytic activity^32^. Carbonylation increases, S-nitrosylation of Cys102 reduces CN1 activity. Under diabetic conditions carbonyl-stress is increased, renal NO_2_^-^/NO_3_^-^ concentrations are reduced^32^, and CN1 is post translationally modified, leading to increased renal CN1 activity^32^.

Glutathione and cysteine are common thiols (R-SH) in mammals. Thiol groups are reducing agents present at intracellular concentrations of approximately 5 mmol/l. Glutathione (γ-l-Glutamyl-l-cysteinylglycine) is present in both the reduced (GSH) and the oxidized (GSSG) state, with the former redox state allowing donation of reducing equivalents from the thiol group of cysteine. In healthy cells and tissues, more than 90% of the total glutathione pool is present in the reduced form and the GSH/GSSG ratio is tightly regulated. Reduction in the GSH/GSSG ratio is a common signature of diabetes-related oxidative stress and contributes to protein dysfunction^33^^,^^34^. In light of recent findings showing that renal glutathione concentrations are reduced in diabetic conditions, in this present study the question of whether CN1 activity is influenced by interaction of cysteine residues in CN1 with glutathione was addressed. In addition, the putative role of S-cysteinylation by thiol-containing compounds on CN1 activities was examined along with the underlying mechanism of CN1 regulation, which was investigated by molecular dynamic (MD) simulations.

## Material and methods

### Carnosinase activity

CN1 activity was assayed according to the method described by Teufel and coworkers^1^. Briefly, the reaction was initiated by addition of carnosine to serum carnosinase at pH of 7. The reaction was stopped after defined periods by adding 1% trichloracetic acid (final concentration in the test 0.3%). Liberated histidine was derivatized by adding o-phtaldialdehyde (OPA) and fluorescence was read using a MicroTek late reader (λExc 360 nm; λExc 460 nm). Interaction of OPA with cysteine, GSH or N-acetylcysteine could be excluded. V_max_ values were obtained from Dixon plots using a linear regression program from five different assays. The kinetic parameters were determined by using various concentrations of carnosine, and data fitting was performed according to Michaelis–Menten equation.

### Recombinant CN1 enzyme

Recombinant FLAG-tagged proteins have been purified from CHO supernatant as described previously^32^. Briefly, CHO cells were transfected with expression vector for CN1 wild type. FLAGG-tagged CN1 was secreted in the supernatant and concentrated and washed with TBS. Purity of recombinant enzyme was checked by silver staining. Substitution of cysteine with serine at position 102 and 229 (Mut1^C102S^ and Mut 2^C229S^) were performed as previously described^32^.

### Diabetic mice

Male C57BL/KsJm/Leptdb (db/db) mice (Stock 00062) and their normal normoglycemic herozygous littermates were obtained from Charles River (Sulzfeld, Germany). The mice were treated as previous described^32^. The experimental procedure was approved by the North Stockholm Ethical Committee for Care and Use of Laboratory Animals^21^. Twenty-one-week-old animals were euthanized by carbon-dioxide. The kidneys were removed, immediately homogenized in cold buffer containing 20 mM HEPES, 1 mM ethylene glycol-tetraacetic acid (EGTA), 210 mM mannitol and 70 mM sucrose per gram tissue, pH 7.2. The homogenate was centrifuged at 1500 *g* for 5 min at 4 °C, and the supernatant was kept at −80 °C until analysis^21^. Protein concentration was determined by Bradford Assay.

### Set up of the carnosinase–carnosine complex

The resolved structure of CN1 was retrieved from Protein Data Bank (PDB, http://wwwrcsb.org, Id: 3DLJ) and was initially prepared by removing water molecules and all crystallization additives. Since this structure includes a homodimer, the simulations involved the monomer B which is the monomer with less unresolved gaps (gaps: residues 77–79 and 208–209) and with the higher percentage of residues falling in the allowed regions of the Ramachandran plot (82.23% vs. 81.95%). The included gaps were then filled by using the corresponding segments of the previously reported homology model^35^ and the completed protein was firstly minimized by taking fixed all atoms apart from those included into a 8 Å radius sphere around the inserted segments followed by a minimization with backbone atoms fixed to optimize the overall protein structure preserving the experimental folding.

The so obtained CN1 structure was then utilized in docking simulations to generate the complex with carnosine. In detail, the optimized carnosine structure was built as described in previous studies^36^ and docking calculations were performed by PLANTS, which generates reliable poses by ant colony optimization algorithms^37^. The search was focused into a 10 Å radius sphere around the barycenter of the two zinc ions, the calculations produced 20 poses which were scored by using the ChemPLP score function with speed equal to 1. The so generated best complex was finally minimized by taking fixed all atoms apart from those included into a 10 Å radius sphere around the bound ligand.

### Set up of the cysteinylated complexes and molecular dynamics (MD) simulations

The optimized carnosinase–carnosine complex was used to generate the two corresponding cysteinylated complexes by manually adding the cysteine structure on Cys102 and Cys229. Since the two cysteine residues are sufficiently exposed this manual modification was performed without difficulty. In this way generated cysteinylated proteins were refined by an energy minimization taking fixed all atoms apart from those included into a 10 Å radius sphere around the inserted cysteine in order to optimize its arrangement on the protein surface.

The minimized structures were neutralized by adding 21 sodium ions by using the program SODIUM (http://www.ks.uiuc.edu/Development/MDTools/sodium/) as implemented in the VEGA suite of programs^38^ and the neutralized structures underwent a preliminary minimization keeping fixed the backbone atoms to optimize the relative position of the sodium ions. The neutralized structures were then inserted into a 70 Å × 100 Å × 70 Å water box containing about 10,200 water molecules. The hydrated systems underwent energy minimization to optimize the relative pose of the solvent molecules and the so optimized structures underwent 20 ns molecular dynamics (MD) simulations with the following characteristics: (a) Newton’s equation was integrated using the r-RESPA method (every 4 fs for long-range electrostatic forces, 2 fs for short-range non-bonded forces, and 1 fs for bonded forces); (b) the simulation space was stabilized by introducing the Periodic Boundary Conditions (70 Å × 100 Å × 70 Å); (c) the long-range electrostatic potential was treated by the Particle Mesh Ewald summation method (70 × 100 × 70 grid points); (d) the temperature was maintained at 300 ± 10 K by means of the Langevin’s algorithm; (e) Lennard-Jones (L-J) interactions were calculated with a cutoff of 10 Å and the pair list was updated every 20 iterations; (f) a frame was stored every 10 ps, to yield 2000 frames. The simulations were carried out in two phases: an initial period of heating from 0 K to 300 K over 300,000 iterations (300 ps, i.e. 1 K/ps) and the monitored phase of 20 ns. The mentioned minimizations were performed using the conjugate gradient algorithm until the r.m.s. gradient was smaller than 0.01 kcal mol-1 Å-1. All calculations were carried out by NAMD 2.7 with the force-field CHARMm v22 and Gasteiger’s atomic charges^39^.

### Statistical analysis

A minimum of three independent experiments were performed in duplicates and more. Data are given as mean ± SD. For comparison of three of more groups a one-way analysis of variance was performed, followed by *post hoc* analyses using Tukey´s test. Differences were considered significant at *p* < .05.

## Results

### Effect of thiol-containing compounds on CN1 activity

#### Human serum carnosinase activity

**Figure 1.**
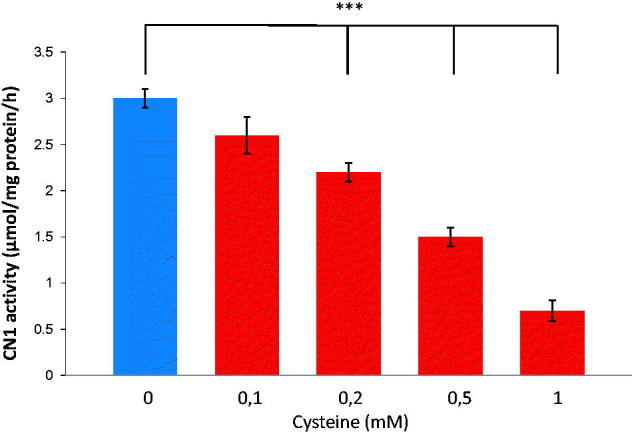
Dose-dependent effect of cysteine on recombinant CN1 activity. Levels of 0.2 mM cysteine and higher, resulted in significantly reduced CN1 activity (*n* = 8, *p* < .005).

The activity of recombinant CN1 (rCN1), produced in CHO cells, was concentration-dependently reduced by reduced glutathione (GSH), cysteine, N-acetylcysteine (Table 1) and cysteine (Figure 1 shows the effect of cysteine). Addition of 1 mM thiol-containing substrate, CN1 activity was reduced, whereas Km values were hardly affected (Table 1), indicating an allosteric inhibition. Reduced CN1 activity was achieved by adding 0.2 mM cysteine (*p* < .001), 0.6 mM GSH (*p* < .01) or 0.4 mM N-acetylcysteine (*p* < .01). Efficiency of degradation of l-glutamic acid (5.2 ± 0.2 µmol/mg/h/mM; *p* = ns) and glycine (5.8 ± 0.4 µmol/mg/h/mM; *p* = ns), components of glutathione and GSSG, had not effect on CN1 activity (5.2 ± 0.3 µmol/mg/h; *p* = ns) with *K*_m_ values (Table 1) of comparable value.

**Table 1.** Catabolic rate of serum CN1 in the presence of inhibitors (*n* = 5).

	*V*_max_ (µmol/mg/h)	*K*_m_ (mM)	Efficiency (*V*_max_/*K*_m_)
Control	4.2 ± 0.4	0.8 ± 0.1	5.2 ± 0.2
Cysteine (1 mM)	1.8 ± 0.2*	1.0 ± 0.2	1.6 ± 0.2*
N-acetylcysteine (1 mM)	2.4 ± 0.2*	1.1 ± 0.3	2.0 ± 0.3*
GSH (1 mM)	2.9 ± 0.4*	0.9 ± 0.2	3.2 ± 0.4*
GSSG (1 mM)	4.1 ± 0.5	0.9 ± 0.3	4.6 ± 0.4
l-Glutamic acid (1 mM)	4.2 ± 0.5	0.8 ± 0.2	5.2 ± 0.3
Glycine (1 mM)	4.1 ± 0.4	0.7 ± 0.3	5.8 ± 0.4

Efficiency of recombinant CN1 activity were calculated by the ratio of *V*_max_ and *K*_m_.

* *p* < .05 compared to control.

#### Role of cysteinylation

Substitution of both rCN1 cysteine residues at position 102 (Mut1^C102S^) and 229 (Mut2^C229S^) showed that cysteine at position 102 but not at position 229 is mandatory for regulation of CN1 activity by thiols. The efficiency for carnosine degradation of Mut1^C102S^ was not influenced by the addition of cysteine. The addition of cysteine to rCN1 Mut2^C229S^ significantly reduced CN1 efficiency (1.8 ± 0.6 µmol/mg/h/mM; *p* < .05) compared to the catalytic efficiency of rCN1 in thiol-free medium (5.2 ± 0.2 µmol/mg/h/mM). The inhibitory effect on catalytic efficiency by cysteine is comparable for Mut2^C229S^ and rCN1 (1.6 ± 0.2 µmol/mg/h/mM).

#### Carnosinase activity in renal kidney tissue of diabetic mice

**Figure 2.**
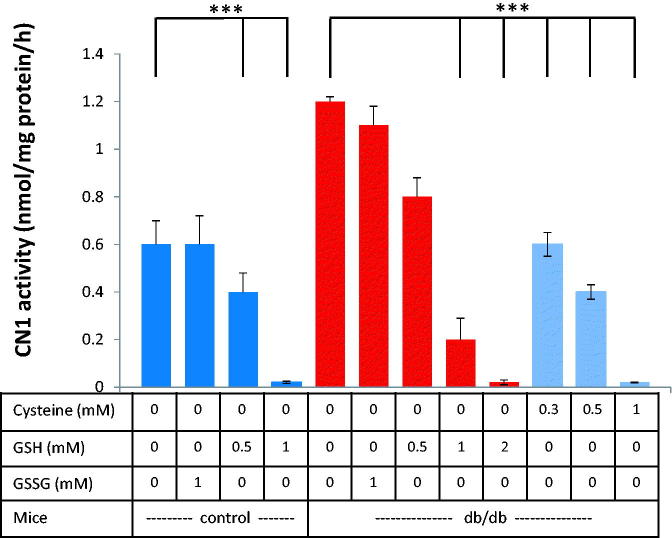
Dose-dependent effect of reduced glutathione (GSH) on renal CN1 activity of db/db mice and controls (at 25 weeks age). Addition of GSH, but not the addition of GSSG, reduced CN1 activity dose-dependently. 0.5 mM GSH significantly lowered CN1 activity for control and control mice (*n* = 8; *p* < .005).

CN1 activity in kidney tissue of db/db mice and controls at age 21 weeks was dose-dependently inhibited by cysteine or GSH, but not by GSSG (data not shown) (Figure 2). Renal CN1 activity was higher in diabetic (db/db) mice as compared to wild-type littermates (1.2 ± 0.2 vs. 0.6 ± 0.4 µmol/mg/h; *n* = 3, *p* = .001). Levels of 0.3 mM Cysteine or 0.5 mM GSH and higher decreased CN1 activities in diabetic and control mice. The addition of 1 mM GSH completely abolished CN1 activity in control mice and decreased CN1 activity reduced by more than 80% in db/db mice (0.2 ± 0.08 µmol/mg/h).

### The carnosinase–carnosine complex

**Figure 3.**
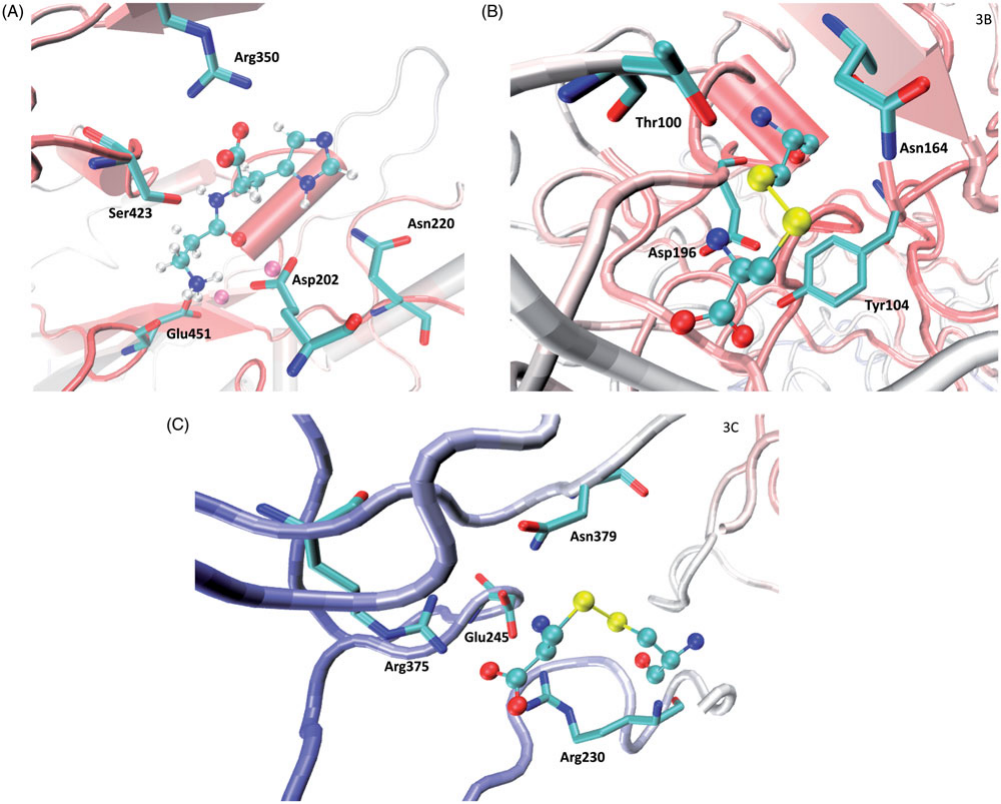
Main interactions stabilized with carnosinase by: (A) carnosine within the catalytic pocket as computed by initial docking simulations; (B) Cys102-cysteinylated residue as derived at the end of the MD simulation; (C) Cys229-cysteinylated residue as derived at the end of the MD simulation.

Figure 3(A) shows the putative complex between carnosinase and its natural substrate carnosine. The complex appears to be vastly stabilized by the key ion-pair between the carnosine carboxyl group and Arg350, while a Zn^2+^ ion polarizes the carbonyl group thus facilitating water-mediated hydrolysis. The carnosine amino group is engaged in the ionic network involving the Zn^2+^ ions and in particular, approaches Asp202 and Glu451. Ser423 seems to act as a bridge stabilizing H-bonds with both carboxyl and amino groups. The imidazole ring of carnosine is inserted into a rather hydrophobic cavity where it can stabilize π–π stacking with His452 plus weak H-bonds with Gln110 and Asn220. Although the contacts elicited by the imidazole appear somewhat marginal, they are, however, suitable to render the enzyme selective for histidine-containing dipeptides.

### Effect of cysteinylated residues

Besides analyzing the effects of the cysteinylated residues on carnosine binding, we investigated the effects of modifications of the regions around the cysteinylated residues through MD simulations. Figure 3(B) shows the interactions elicited by S-cysteinylated Cys102 at the end of the simulation. Specifically, the cysteinyl ammonium head is engaged in a clear ion-pair with Asp196, while the carboxyl group elicits H-bonds with Ser193 and Tyr104. The disulfide bridge is involved in H-bonds with Thr100 and Asn164 plus extended sulphur-π interactions with Tyr104. Thus, S-cysteinylation induces the key approach of Asp196 and Tyr104 in a pose stably conducive to the above-mentioned contacts. Similarly, Figure 3(C) depicts the contacts stabilized by the cysteinylated Cys229 residue at the end of the MD run and reveals the rich set of ion pairs that the charged termini of the cysteinyl residue stabilize with Arg230, Glu245, Asp249 and Arg375. In contrast, the disulfide bridge elicits only a weak H-bond with Asn379 plus hydrophobic contacts with Val227 and Val364. The comparison of the first and last structures coming from the MD simulation reveals the marked approach of the above-mentioned ionized side chains which progressively focusing on the cysteinyl ionized termini.

The primary objective of the reported MD simulations involved the analysis of the effects of S-cysteinylation on carnosine binding with a view to explain the inhibiting effect ascribable to the sole modification of Cys102. The key interactions stabilizing the carnosinase–carnosine complex were monitored during the two MD runs revealing the key differences between the two cysteinylated forms which are in line with the reduced catalytic efficacy of the S-cysteinylated Cys102 form. Indeed, Figure 4(A) compares the time-dependent profile of the distance between the carnosine carboxyl terminus and Arg350 as generated by the two MD runs and suggests that this key ionic contact is stably and similarly retained throughout both MD runs. In contrast, Figure 4(B) shows the corresponding distance profiles for the catalytically crucial interaction between the carnosine carbonyl group and the Zn^2+^ ions and reveals notable differences between the two simulated modifications. Indeed, such a contact is stably conserved in the S-cysteinylated Cys229 form, while it appears to be clearly weakened in the S-cysteinylated Cys102 form as confirmed by the distance average (as computed over the entire MD run) shifting from 4.26 Å to 5.98 Å. Taken together, the weakened contacts stabilized by carnosine in the S-cysteinylated Cys102 form is reflected by a greater mobility of the ligand as shown in Figure 4(C) and confirmed by a RMSD average which shifts from 1.98 Å to 3.18 Å. The different interactions can be also evaluated by the pair interaction calculations as implemented in Namd which computes the interaction energy, as decomposed into ionic plus van der Waals terms, by applying the same parameters with which the MD simulations were performed. As shown in Figure 4(D), the major difference between the two analysed simulations involves the ionic contacts which reveal a clear (albeit not very marked) weakening induced by the S-cysteinylation of Cys102. The van der Waals term appears to be comparable or at most S-cysteinylation of Cys102 induces a slight strengthening of such contacts probably as they tend to counteract the missing polar contacts even though the overall interaction energy confirms the destabilizing effects induced by S-cysteinylation of Cys102 (results not shown).

Figure 4.Destabilizing effects of Cys102-S-Cysteinylation on carnosinase–carnosine complex as assessed by comparing the dynamic behaviour in the two performed MD runs of (A) the distance between the carnosine’s carboxyl group and Arg350; (B) the distance between the carnosine’s carbonyl group and the Zinc ion; (C) the carnosine mobility as evaluated by rmsd values computed by considering only the carnosine atoms; (D) the ionic interaction energy as computed by Namd2.7.

**Figure:**
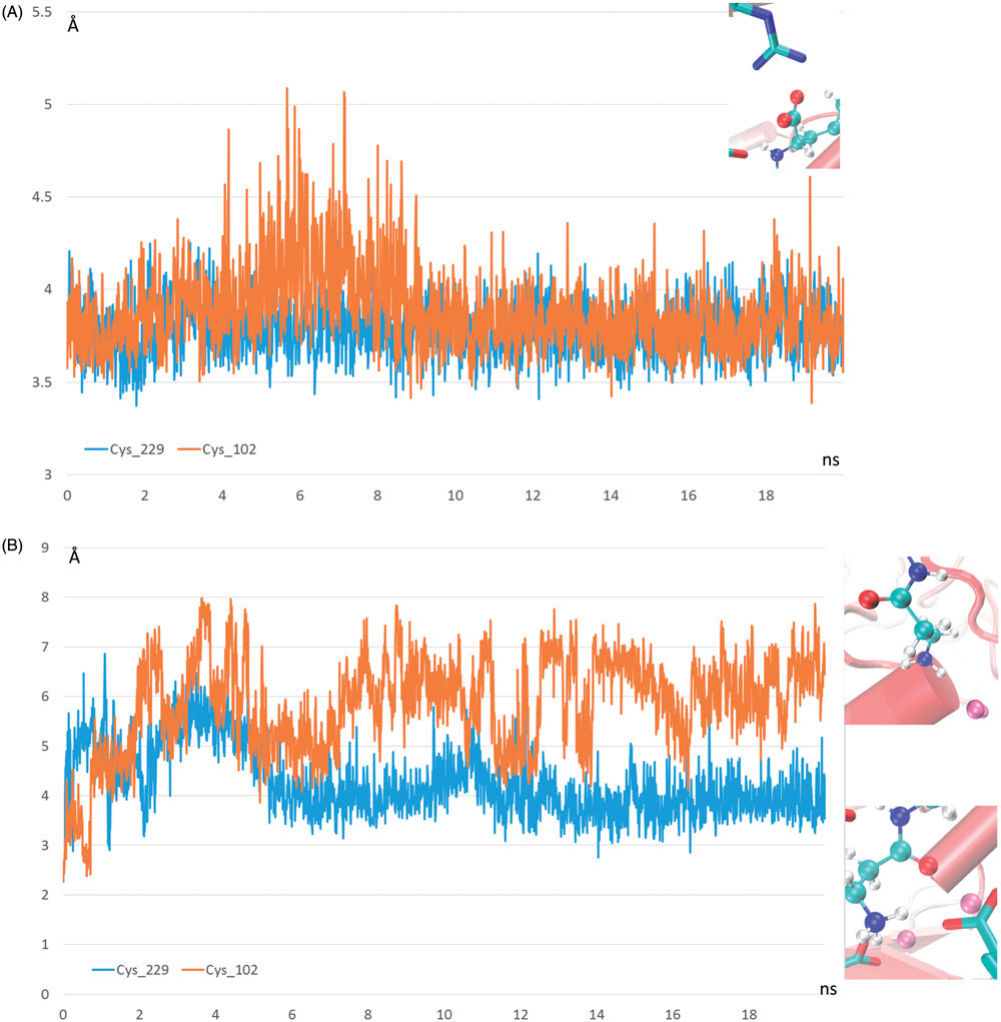

**Figure:**
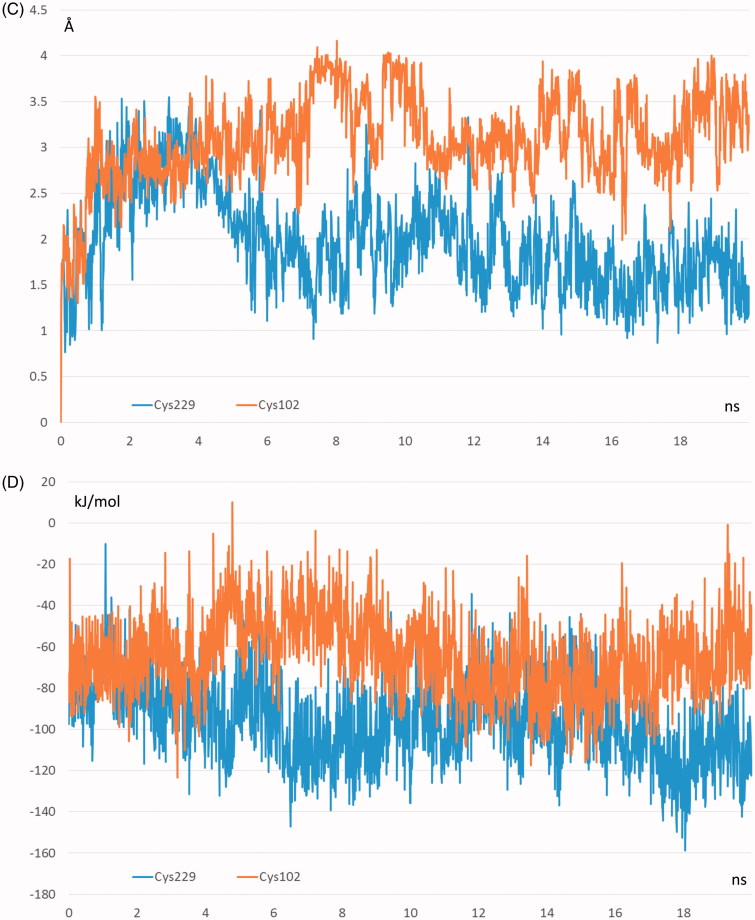

## Discussion

Since the relevance of carnosine in diabetes^2^^,^^18^, cancer and neurological diseases^40–42^ is well described, understanding the molecular basis of CN1 regulation and its effect on carnosine levels is essential to provide potential novel therapeutic approaches. In diabetic mice and rats, decreased carnosine content was found in retina, kidney and liver^24^^,^^26^^,^^43^ whereas renal CN1 was increased^21^. The increase of renal CN1 activity of diabetes is caused by post-translational modifications, i.e. increased carbonylation and reduced S-nitrosylation, i.e. the covalent binding of NO to cysteines^32^. CN1 activity is regulated by modifications of cysteine at position 102, while cysteine at position 229 was shown to be irrelevant for enzyme function^32^. The active site of CN1 does not contain cysteine residues, but Cys102 is located in the same β-strand as His106. We now demonstrate a novel mechanism of Cys102 modification, inhibiting CN1 activity. Thiol-containing compounds, such as GSH, cysteine and N-acteylcysteine, reduce CN1 activity, whereas thiol-free components such as l-glutamic acid and glycine and oxidized glutathione, have no effect on CN1 activity. Cysteine substitution in recombinant CN1 demonstrated the relevance of cysteinylation of cysteine at position 102 on catalytic efficiency. In renal tissue homogenates of diabetic mice, the addition of GSH normalized renal CN1 activity, indicating that the ratio of reduced to oxidized glutathione is important for CN1 regulation. In renal tissue of diabetic mice GSH concentrations are inversely related to CN1 activity^32^.

In biological systems, thiols are found in cysteine and derived molecules of low and high molecular weight at millimolar concentrations^44^. Cysteine plays protective roles in maintaining the redox state by S-cysteinylation, a reversible reaction which shields protein thiols by preventing their irreversible oxidation to sulfonic acids^45^. Due to its strong nucleophilicity, compared with other amino acids, cysteine is more prone to oxidation by ROS^46^. Common reversible modifications of cysteine include formation of sulfenic acid (SOH), S-nitrosylation (SNO), S-glutathionylation (SSG), S-palmitoylation, and the formation of disulphide bonds^47^. These reversible post-translational modifications (PTMs) have important biological roles and help maintain homeostasis by preventing the formation of irreversible oxidative modifications [e.g. sulfinic (SO2H) and sulfonic acid (SO3H)]^48^. Furthermore, these modifications are critical for cellular signalling. Metabolic imbalance resulting from reversible or irreversible PTMs of cysteine residues can lead to cellular damage. Specifically, thiol-based redox regulation is important in metabolism and dysregulated thiol redox homeostasis has been implicated in aging and diseases, such as cancer, cardiovascular, neurodegenerative diseases and diabetes^46^. Thus, better understanding of the landscape of the thiol redox proteome can give insight into biochemical events that occur in disease, and may lead to potential biomarkers for disease diagnosis and therapeutic interventions.

The present study proposes a mechanism by which increased CN1 activity can be inhibited in diabetes as well as during aging, in order to counteract the decreasing carnosine level^49^, which depends on the cysteine levels and redox state. Inhibition of CN1 activity by compounds such as N-acetylcysteine, which are licensed for liquefaction of the mucus in bronchopulmonary disease and as antidote, e.g. in case of paracetamol intoxication, should provide the option to increase systemic carnosine concentrations by oral supplementation. Augmentation of systemic histidine dipeptide levels by exogenous carnosine in humans potentially exerting protective actions as repeatedly described in rodents lacking serum CN1, up to now where prevented by rapid degradation by CN1. S-cysteinylation, which usually has the sole objective of protecting key protein thiol groups, is able to allosterically and potently reduce CN1 enzymatic activity and thus should increase the plasma levels of carnosine which can actively participate in the overall antioxidant defence and cytoprotection^18^. To the best of our knowledge, this is the first report documenting that S-cysteinylation has not only a protective effect but also a regulatory allosteric role. Previous studies suggested that it can influence protein dimerization as seen in Cu^2+^/Zn^2+^-containing superoxide dismutase-1^50^. However, such a modulatory mechanism might be shared by other S-cysteinylated proteins. Interestingly, such a mechanism induces a transient and partial enzymatic inhibition which would be largely favourable compared to an irreversible and/or complete inhibition especially for those tissues where CN1 regulates the release of GABA from homocysteine. In other words, an allosteric inhibition seems to be well suited for CN1 since it allows to finely increasing the carnosine level without completely abolishing its enzymatic activity which might lead to the accumulation of histidine-containing dipeptides.

S-cysteinylation reduces the maximum rate of the reaction without changing the apparent binding affinity for carnosine (*K*_m_ value). This indicates the mechanism of noncompetitive inhibition, involving reversible binding to an allosteric site. Allosteric ligands influence activity by binding to sites that are topographically distinct from orthosteric binding sites^51^. Allosteric sites allow effectors to bind to the protein, which often results in a conformational change involving protein dynamics. The underlying putative complex between CN1 and carnosine by MD simulation is in agreement with that recently proposed by Pavlin and coworkers^28^. The comparison revealed that the most remarkable conformational shift characterizing the S-cysteinylated form of Cys102 is that involving Tyr104 which stably contacts a Zn^2+^ ion in the simulation involving the S- cysteinylated Cys229-containing enzyme, while in the S- cysteinylated Cys102 form it leaves the Zn^2+^ ion to approach the cysteinylated residue. Such a conformational change induces a rearrangement of the negatively charged residues surrounding the Zn^2+^ ions which in turn causes a partial shift of the carnosine ammonium head with the consequent distancing of the carbonyl group. Thus, the presented results can account for the partial inhibition of the catalytic activity of the S-cysteinylated Cys102 which yet retains part of its catalytic activity as suggested by the overall limited differences detected between the two MD runs.

## Conclusions

In conclusion, we provide evidence for a novel mechanism of CN1 regulation. Kinetic parameters and MD simulations revealed that inhibition by thiol-containing compounds is due to allosteric interactions through S-cysteinylation. Recent research has pinpointed allosteric interactions as a useful tool to modulate receptor function in ways that cannot be achieved by ligands that bind to an orthosteric site^52^. Therefore, allosteric ligands can present therapeutic advantages over orthosteric ligands. Inhibition of circulating and tissue CN1, resulting in higher carnosine levels, may represent a valuable therapeutic strategy for mitigation of complications associated with diseases such as diabetes mellitus.
